# The comparison of sex differences in depression-like behaviors and neuroinflammatory changes in a rat model of depression induced by chronic stress

**DOI:** 10.3389/fnbeh.2022.1059594

**Published:** 2023-01-04

**Authors:** Juan Xia, Haoyin Wang, Cai Zhang, Baiping Liu, Yuyu Li, Kangwei Li, Peng Li, Cai Song

**Affiliations:** ^1^Research Institute for Marine Drugs and Nutrition, College of Food Science and Technology, Guangdong Ocean University, Zhanjiang, China; ^2^Laboratory of Hematologic Diseases, Affiliated Hospital of Guangdong Medical University, Zhanjiang, China; ^3^Stem Cell Research and Cellular Therapy Center, Affiliated Hospital of Guangdong Medical University, Zhanjiang, China; ^4^Marine Medicine Research and Development Center of Shenzhen Institutes, Guangdong Ocean University, Shenzhen, China

**Keywords:** depression, chronic stress, inflammation, neurotrophic factors, sex difference

## Abstract

**Background:**

Clinical prevalence of major depression is higher in women than men, while the psychoneuroimmunological mechanisms underlying the differences between the two sexes are not fully understood.

**Methods:**

The present study explored sex differences in the behaviors and depressive pathological mechanisms induced by chronic unpredictable mild stress (CUMS). Depression- and anxiety-like behaviors were assessed by the sucrose preference test (SPT), force swimming test (FST), open field test (OFT), and elevated plus-maze (EPM). The enzyme-linked immunosorbent assay (ELISA) was used to measure cytokine concentrations, high-performance liquid chromatography (HPLC) was used to measure monoamine neurotransmitters and metabolite contents, and real-time quantitative PCR (qPCR) and western blotting (WB) were used to measure glial parameters in the hippocampus.

**Results:**

Under control conditions, female rats exhibited shorter immobility times in the FST, lower interferon (IFN)-γ, and interleukin (IL)-4 levels in the hippocampus, lower norepinephrine (NE) and homovanillic acid (HVA), and higher p75 and glial-derived neurotrophic factor (GDNF) expression than male rats. CUMS markedly reduced rat body weight gain, sucrose preference, locomotor activity, number of entries into the central zone and rearing in the OFT, as well as the number of entries into and time spent in open arms of the EPM; however, CUMS increased the immobility times of the rats of both sexes in the FST. Interestingly, more pronounced changes in sucrose preference and locomotor activity were observed in female rats than in males. Consistently, CUMS-increased glucocorticoid concentration, M1 microglial marker CD11b, and peripheral IL-1β and IL-4, while decreased hippocampal IL-10, serotonin (5-HT), dopamine metabolite 3,4-dihydroxyphenylacetic acid (DOPAC), and norepinephrine metabolite 3-methoxy-4-hydroxyphenylglycol (MHPG) were more significant in females than in males.

**Conclusion:**

These data revealed possible mechanisms by which females suffer more depression than males at least in a stressful environment.

## Introduction

The macrophage and T-lymphocyte hypothesis of depression (Smith, 1991; Maes et al., 1995) points out that excessive production of proinflammatory cytokines can stimulate the hypothalamic-pituitary-adrenal (HPA) axis to overproduce glucocorticoids (GCs), which disrupts the GC feedback system and induces HPA axis dysfunction (Turnbull and Rivier, 1995). Increased permeability of the blood–brain barrier induced by inflammatory response promotes the entry of inflammatory factors into the brain (Yang et al., 2019), ultimately leading to neuroinflammation through microglial activation (Moraes et al., 2021). Peripheral and central inflammation can also activate the indoleamine-2,3-dioxygenase (IDO) pathway, which in turn increases central neurotransmitter metabolism, thus reducing the synthesis of monoamine neurotransmitters (Christmas et al., 2011). Epidemiological studies in humans have consistently shown that the incidence of depression in women is approximately twice as high as that in men (Kessler, 2013). Moreover, there was a significant sex difference in molecular signatures in patients with depression (Seney et al., 2018), and the manifestations of major depressive disorder in women are more complex, and women exhibit greater severity, more symptoms, and different symptomatology (Cavanagh et al., 2017). Compared to that in men, the HPA axis in women is more sensitive to stress, and female hormones, such as ovarian hormones, may interact with GCs during stress. Both of these mechanisms may accelerate the onset of depression and exacerbate symptoms. However, according to macrophage/T-lymphocyte theory, neuroinflammatory differences between male and female depressed patients have not been fully understood. Herein, the present study aimed to explore sex differences in a rat model of depression.

Our laboratory previously established a stable model of depression that is induced by CUMS. After exposure to chronic unpredictable mild stress (CUMS), these rats exhibit anxiety (Zhang et al., 2019a) and depression-like behavior (Zhang et al., 2019b), the mechanism of which mainly involved increasing concentrations of glucocorticoids, imbalance between microglia M1 proinflammatory phenotype and M2 anti-inflammatory phenotype polarization, and decreasing expression of the astrocyte marker glial fibrillary acidic protein (GFAP), the brain-derived neurotrophic factor (BDNF) and the tyrosine kinase B receptor (TrkB). Thus, the present study evaluated the behavioral differences underlying possible psychoneuroimmunological mechanisms between male and female rats in the control and CUMS exposing condition.

## Materials and methods

### Animals

Thirty-two male (280–350 g) and 32 female 9 weeks Sprague–Dawley (SD) rats (195–230 g) were purchased from the Animal Experiment Center, Southern University of Science and Technology, Shenzhen, China. Two rats of the same sex were housed in a standard polycarbonate shoebox cage. All rats were free to access to food and water. The animal colony was maintained at 23 ± 1°C and 50 ± 10% relative humidity under a 12/12 h light/dark cycle. Upon arriving, rats were allowed to acclimate to the laboratory environment for 1 week prior to experimental procedures. Studies involving animals were performed with three Rs principles (Replace, Reduce, and Refine) according to internationally accepted standards. The animal experiment protocol was performed in accordance with the International Guiding Principles for Animal Research provided by the International Organizations of Medical Sciences (CIOMS) Council and was approved by the Bioethics Committee of Guangdong Ocean University.

### Experiment procedure

**FIGURE 1:**
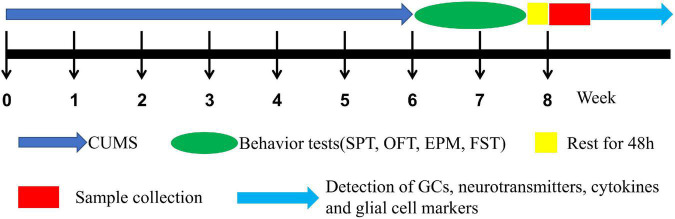
The experimental procedure.

Rats were randomly assigned to 4 groups (*n* = 16) such as (1) male control (CTM), (2) male CUMS (SM), (3) female control (CTF), and (4) female CUMS (SF). As shown in Figure 1, CUMS exposure was 6 weeks and body weight was measured every week. Then, behavioral tests, such as the sucrose preference test (SPT), open field test (OFT) elevated plus-maze (EPM), and force swimming test (FST), were carried out in turn. All the behavioral tests were performed with the observer blind, and the rats were sacrificed two days after the behavioral tests. Serum and hippocampal samples were collected and stored at –80°C for further measurements.

### CUMS exposure paradigms

The establishment of the CUMS strategy referred to the method reported by Banasr et al. (2007) with minor modifications. The stressors for CUMS are shown in Table 1. To make the procedure unpredictable, two different stressors were randomly applied each day, and the same stressor was not allowed for three consecutive days.

**TABLE 1:** The stressors for CUMS in this study.

Stressor	Duration	Stressor	Duration
Restraint in a tube	1 h	Stroboscopic light overnight	12 h
Swimming in 18°C water	10 min	Lonely overnight	12 h
Crowed overnight	12 h	Peculiar smell overnight	12 h
Lighting overnight	24 h	Water or food deprivation	12 h
Light cycle	3 h	Stress under 4°C	1 h
Mouse cage overnight	12 h	Wet bedding overnight	12 h
Cage tilting at 45°	12 h		

### Sucrose preference test

The SPT, for assessing anhedonia-like behavior, was carried out as described previously (Willner et al., 1987). Before the test, rats were acclimated to 1% sucrose solution (w/v). On day 1, rats were offered two bottles of 1% sucrose water to consume. On day 2, animals were offered one bottle of 1% sucrose water and one bottle of fresh water. On day 3, they were individually housed in cages, with food and water deprivation for 24 h. On day 4, each rat was offered one bottle of pre-weighed 1% sucrose water and one bottle of pre-weighed fresh water. Then, the consumption of sucrose water and fresh water was weighed after 4 h. The sucrose preference (SP) was calculated based on the formula: SP (%) = sucrose intake/(sucrose intake + water intake) × 100%.

### Open field test

The OFT has been widely used to identify the spontaneous activity of rodents (Hu et al., 2017). A round open field apparatus (100 cm in diameter, 50 cm in height), in which the walls and floor were painted white, was used, and a 60-W white bulb was positioned above the center of the apparatus (Yan et al., 2021). For the test, each rat was placed with its head toward the walls, and the behavioral activity was recorded for 3 min by the SuperMaze behavior analysis system (version 2.0, Shanghai Xinruan Information Technology Co., Ltd., Shanghai, China).

### Elevated plus-maze test

The EPM has been widely used to measure anxiety-like behaviors in rodents (Kraeuter et al., 2019). In general, the maze is plus-shaped, consists of two enclosed arms and two open arms, and is elevated 60 cm above the floor. A 40-W bulb was positioned 1.5 m above the center of the maze as previously described (Li et al., 2021). During the test, each rat was placed at the center facing the same open arms. The number of entries and the time spent in the open and closed arms were recorded for 5 min using the SuperMaze behavior analysis system (version 2.0, Shanghai Xinruan Information Technology Co., Ltd., Shanghai, China).

### Forced swim test

The FST was used to evaluate the despair-like behaviors of rats while swimming in a vertical plastic cylinder (20 cm in diameter, 50 cm in height) containing 30 cm of water that was maintained at 25°C. Twenty-four hours before the FST, the rats were forced to swim for 10 min. During the following 5 min, the immobility time of each rat was recorded and analyzed by VisuTrack animal behavior analysis software (Shanghai Xinruan Information Technology Co., Ltd., Shanghai, China).

### Blood sample and hippocampus tissue separation

After coagulation at room temperature, blood samples were centrifuged at 1,800 rpm for 10 min. The serum was transferred to a new centrifuge tube and frozen at –80°C for cryopreservation. Rat brains were collected, and the hippocampi were rapidly separated on ice, frozen in liquid nitrogen, and transferred to a –80°C freezer for further measurements.

### Corticosterone and cytokines detection

The corticosterone levels in the rat serum were measured using an enzyme-linked immunosorbent assay (ELISA) kit (Nanjing Jiancheng Bioengineering Institute, Nanjing, China) according to the manufacturer’s instructions. Moreover, the levels of inflammatory factors, including the proinflammatory factor interleukin (IL)-1β, interferon gamma (IFN)-γ, and the anti-inflammatory factors IL-4, IL-10, and transforming growth factor (TGF)-β, were also measured by ELISA purchased from Beijing Dongge Boye Biotechnology Co., Ltd. The hippocampal tissues were homogenized in pH 7.4 PBS at a ratio of 1:9 by an electric homogenizer and centrifuged at 2,000 rpm for 15 min at 4°C. The supernatants were stored at –80°C.

### Neurotransmitter and metabolite concentrations analyzed by high-performance liquid chromatography

The levels of neurotransmitters and metabolites were measured according to a previously described method (Peng et al., 2020). Hippocampal tissues were homogenized in ice-cold 0.60 mol/L perchloric acids supplemented with 50 mmol/L Na_2_EDTA, and 100 ng isoproterenol was added as an internal standard. After centrifugation twice at 14,000 rpm for 15 min at 4°C, the supernatants were collected and filtered through a 0.45 μm membrane. Then, the pH was adjusted to 3.8 with 1 mol/L sodium acetate, and the samples were stored at 80°C until further study. For high-performance liquid chromatography (HPLC) analysis, 20 μl of the sample solutions were injected into the HPLC system with fluorescence detection (Agilent, Santa Clara, CA, USA) and separated by a C18 reverse-phase column (4.5–150 mm) (Agilent, Santa Clara, CA, USA) with a mobile phase containing sodium acetate and citric acid. The mobile phase was prepared as follows: 0.1 mol/L sodium acetate and 0.1 mol/L citric acids were mixed in a 10:9 ratio, and the PH was adjusted to 3.5 using 0.1 mol/L sodium citric buffer. Then, methanol was added at a ratio of 85:15 with sodium octane sulfonate (100 mg/L) and Na_2_EDTA (5 mg/ml).

### Reverse transcription and real-time quantitative polymerase chain reaction

Total RNA was extracted from hippocampal tissues with TRIzol reagent (15596026, Invitrogen, USA) according to the manufacturer’s instructions. First, 100 mg of hippocampal tissue was homogenized in TRIzol reagent and incubated at room temperature for 10 min. Subsequently, 200 μl of chloroform was added, vortexed for 30 s, and then incubated at room temperature for 10 min. After centrifugation, the supernatant was incubated with an equal volume of isopropanol at room temperature. Finally, the RNA precipitate was washed with 75% ethanol and dissolved in diethyl pyrocarbonate (DEPC) water. After measuring the concentration and purity of the extracted RNA, 1 μg of total RNA was reverse transcribed into cDNA by HiScript II Q RT SuperMix for qPCR (+ gDNA wiper) (R223-01, Vazyme, China) and qPCRby ChamQ SYBR qPCR Master Mix (Q311-02, Vazyme, China) on a LightCycler 96 detection system. The PCR conditions were as follows: an initial denaturation step at 95°C for 10 min, followed by 40 cycles of 95°C for 10 s and 60°C for 30 s. The sequences of the primers specific to the target genes are listed in Table 2. The mRNA expression levels of the target genes were determined by the 2^–ΔΔct^ method and normalized to the β-actin expression levels in the same sample.

**TABLE 2:** The primer sequences of target genes.

Genes	Forward primer	Reverse primer
BDNF	F:5′-CAAAAGGCCAACTGAAGC	R:5′-CGCCAGCCAATTCTCTTT
TrKB	F:5′-CACACACAGGGCTCCTTA	R:5′-AGTGGTGGTCTGAGGTTGG
P75	F:5′-TGCTCCATTTCCATCTCAG	R:5′-GATAGGTCCGTAATCCTCTTC
CD11b	F:5′-CAAGGAGTGTGTTTGCGTGT	R:5′-AGAAGGCTCGGACAACTGAG
GFAP	F:5′-CCAAGATGAAACCAACCT	R:5′-CGCTGTGAGGTCTGGCTT
GDNF	F:5′-AACCAAGGAGGAACTGAT	R:5′-CGGAATGCTTTCTI AGGAT
GFR-α1	F:5′-CGAGACATCTTGCTTACT	R:5′-CTGAACCTGAACATTGAAG
GFR-α2	F:5′-TGTCACCTCTTCCTCTTG	R:5′-GCTATACTACAGGCACCAT
β-Actin	F:5′-GTCGTACCACTGGCATTGTG	R:5′-CTCTCAGCTGTGGTGGTGAA

### Western blotting

The hippocampus samples were homogenized on ice in cold radioimmunoprecipitation assay (RIPA) lysis buffer supplemented with 1 mM phenylmethanesulfonyl fluoride (PMSF) protease inhibitor, and the lysates were centrifuged at 4°C, and 12,000 rpm for 5 min. The BCA protein assay kit (Beyotime Biotechnology, Nantong, Jiangsu, China) was used to determine the total protein concentration of each sample. The proteins of each sample were separated by 12% sodium dodecyl sulfate–polyacrylamide gel electrophoresis (SDS–PAGE) and transferred onto polyvinylidene fluoride (PVDF) membranes. Subsequently, the membranes were blocked with 5% skim milk dissolved in Tris-buffered saline for 1 h and incubated with primary antibodies overnight. The primary antibodies included antibodies against BDNF (1:1,000, ab108319, Abcam, Cambridge, UK), P75 (1:1,000, ab52987, Abcam, Cambridge, UK), TrK B (1:1,000, ab18987, Abcam, Cambridge, UK), CD11b (1:1000, ab75476, Abcam, Cambridge, UK), GFAP (1:2,000, sc-33673, Santa Cruz, CA, USA), GDNF (1:1,000, ab176564, Abcam, Cambridge, UK), and β-actin (1:2,000, sc-47778, Santa Cruz, CA, USA). Then, the membranes were washed with TBST three times and incubated with an HRP-labeled secondary antibody for 1 h at room temperature. The bands were detected with an enhanced chemiluminescence (ECL) system and quantified by Image J Software.

## Statistical analysis

GraphPad Prism (GraphPad Software 6) was used. The data are expressed as the mean ± SEM. The data for body weight, behavioral tests, the concentrations of corticosterone, inflammatory factors, neurotransmitters, and glial markers were analyzed by two-way (ANOVA), with stress and sex as two individual factors. Differences between groups were assessed by Tukey’s *post hoc* test, and one-way ANOVA was used for body weight at each time point after two-way repeated measures ANOVA. *P* values less than 0.05 (typically ≤ 0.05) were considered to be statistically significant.

## Results

### Sex differences in body weight gains

**FIGURE 2:**
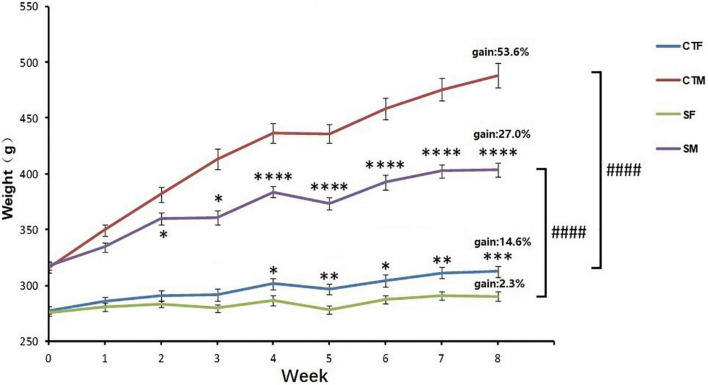
CUMS reduced body weight gain in female and male rats. *p < 0.05, ^**^p < 0.01, ^***^p < 0.001, and ^****^p < 0.0001, vs. control with same sex, ^####^*p* < 0.0001 vs. SM. CTF: female control, CTM: male control, SF: female stress group, SM: male stress group.

The body weight was affected by time (time: *F*_8, 551_ = 72.29, *p* < 0.0001) and CUMS (CUMS: *F*_3, 551_ = 1137, *p* < 0.0001). Moreover, the interaction between time and CUMS significantly affected the body weight gain in both sexes (*F*_24, 551_ = 18.13, *p* < 0.0001). One-way ANOVA analysis of body weight for each time point showed that the body weight was always lower in female rats than in male rats under control conditions (0–8 weeks, *p* < 0.0001), while the percent of body weight gain was also lower in female rats with 14.6 to 53.6% in male rats. CUMS significantly reduced the weight gain in male rats from the 2nd week (*p* = 0.0481) and female rats from the 4th week (*p* = 0.0195), while the weight gain percent was lower in female rats at 2.3% than the males at 27.0%. However, CUMS induced similar body weight loss in both sexes without a difference (36.6% in female rats to 35.3% in male rats) compared with sex-matched control in the 8th week (Figure 2).

### Sex differences in behaviors after exposure to CUMS

**FIGURE 3:**
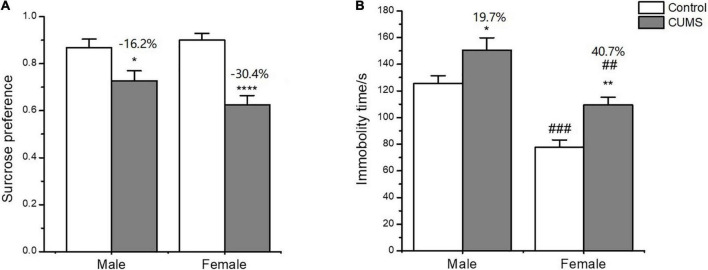
Depressive-like behavior induced by CUMS in SPT and FST of male and female rats. **(A)** Percentage of sucrose consumption in the SPT. **(B)** Immobility time in the FST. *p < 0.05, **p < 0.01, and ****p < 0.0001 vs. control with same sex, ^##^*p* < 0.01, ^###^*p* < 0.001 vs. male in same control or CUMS group.

In the SPT, two-way ANOVA indicated that CUMS significantly affected the sucrose preference (CUMS: *F*_1, 54_ = 32.34, *p* < 0.0001) with no effects of sex difference (sex: *F*_1, 54_ = 0.9554, *p* = 0.3327). CUMS significantly reduced the sucrose preference by 30.4% in female rats and 16.2% in male rats compared with sex-matched control (female: *p* < 0.0001; male: *p* = 0.0356) (Figure 3A).

In the FST, two-way ANOVA indicated that either CUMS (CUMS: *F*_1, 56_ = 12.38, *p* = 0.0009) or sex (sex: *F*_1, 56_ = 24.65, *p* < 0.0001) affected the immobility time. The *post hoc* test showed that the immobility time was shorter for female rats than male rats under control conditions (*p* = 0.0002). CUMS significantly prolonged the immobility time in both sexes compared with sex-matched control (female: *p* = 0.0078; male: *p* = 0.0310), which was more significant in female rats than in male rats (increasing percent of 40.7% and 19.7%, respectively) compared to sex-matched control (Figure 3B).

**FIGURE 4:**
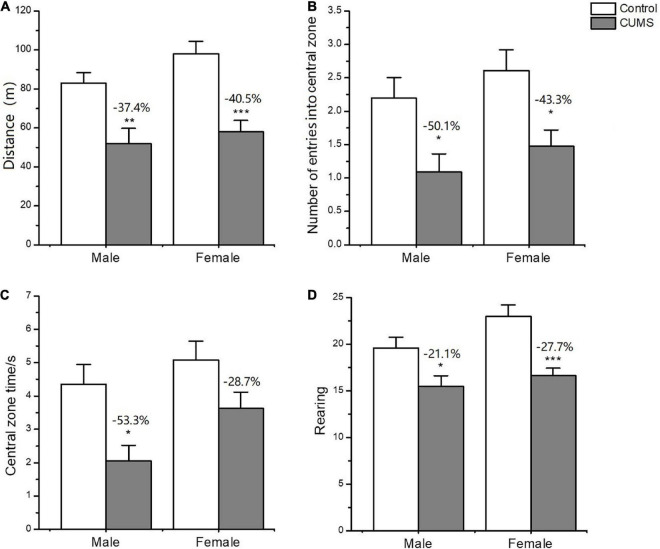
Depressive-like behavior induced by CUMS in OFT of male and female rats. **(A)** The distance moved in OFT. **(B,C)** The number and time spent on entries into central zone. Rearing number in OFT **(D)**. *p < 0.05, **p < 0.01, and ***p < 0.001 vs. control with same sex.

In the OFT, two-way ANOVA indicated that CUMS significantly affected the locomotor activity (*F*_1, 56_ = 33.89, *p* < 0.0001), rearing number (*F*_1, 56_ = 19.74, *p* < 0.0001), number of entries into central zone (*F*_1, 56_ = 16.48, *p* = 0.0002), and time spent on the central zone (*F*_1, 56_ = 13.45, *p* = 0.0005), but there was no sex difference between the males and females. CUMS significantly reduced the locomotor activity (*p* = 0.0036), number of entries into (*p* = 0.0309), and time spent on the central zone (*p* = 0.0122) in male rats at 37.4%, 50.1%, and 53.3%, respectively. Meanwhile, CUMS significantly reduced the locomotor activity (*P* = 0.0001), rearing number (*p* = 0.0008), and number of entries into the central zone (*p* = 0.0266) at 40.5%, 27.7% and 43.3%, respectively, in females compared to the control (Figures 4A-D).

**FIGURE 5:**
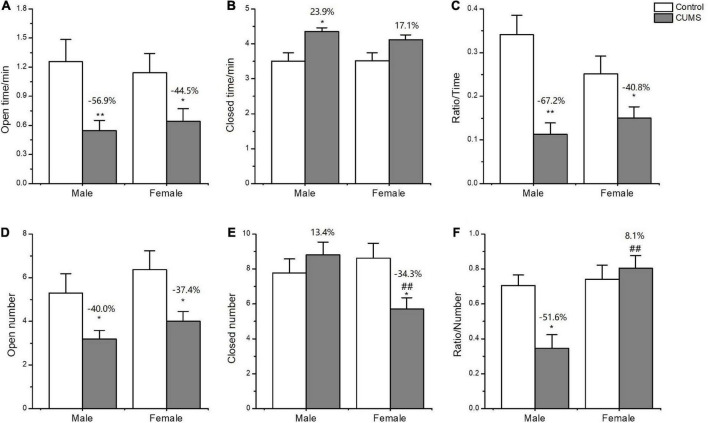
Anxiety-like behavior induced by CUMS in EPM of male and female rats. **(A,B)** Time spent in the open arms and closed arms. **(D,E)** The number of entries into the open arms and closed arms. **(C,F)** The ratio of time spent on open/closed arms and entry numbers of open/closed arms. *p < 0.05, **p < 0.01 vs. control with same sex, ^##^*p* < 0.01 vs. male stress group.

In the EPM test, two-way ANOVA indicated that CUMS significantly affected the number of entries into the open arms (CUMS: *F*_1, 52_ = 18.32, *p* < 0.0001), and time spent on the open arms (CUMS: *F*_1, 52_ = 13.86, *p* = 0.0005) and closed arms (*F*_1, 50_ = 15.68, *p* = 0.0002) for the rats without sex difference. CUMS significantly decreased the number of entries into the open arms (female: *p* = 0.0140; male: *p* = 0.0206) and time spent in the open arms (female: *p* = 0.0316; male: *p* = 0.0037) for the rats of both sexes. However, the interaction between CUMS and sex significantly affected the number of entries into the closed arms (*F*_1, 57_ = 8.520, *p* = 0.0050). CUMS increased male rats’ time spent in the closed arms (*p* = 0.0238) and decreased the female number of entries into the closed arms (*p* = 0.0116), leading to increasing the 8.1% in the ratio of open/closed entry numbers for female rats and decreasing the 51.6% in male rats compared with sex-matched control (Figures 5A-F).

### Sex differences in serum corticosterone levels

**FIGURE 6:**
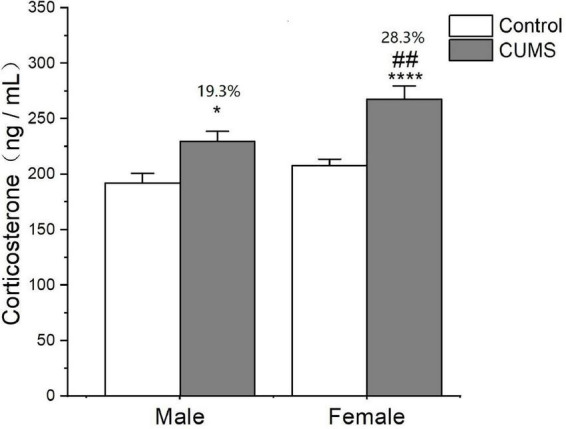
CUMS increased the level of corticosterone in male and female rats. *p < 0.05, ****p < 0.0001 vs. control with same sex, ^##^*p* < 0.01 vs. male stress group.

Two-way ANOVA indicated that either CUMS or sex significantly affected the corticosterone level in the serum (CUMS: *F*_1, 62_ = 27.79, *p* < 0.0001, sex: *F*_1, 62_ = 11.17, *p* = 0.0014). The *post hoc* test showed that there was no sex difference in the serum corticosterone levels observed under control conditions. CUMS significantly increased the corticosterone concentrations in both male and female rats (female: *p* < 0.0001; male: *p* = 0.0235), which increase was much higher in female rats with 28.3% than 19.3% change of male rats compared with sex-matched control (*p* = 0.0081) (Figure 6).

### Sex differences in cytokine concentrations

**FIGURE 7:**
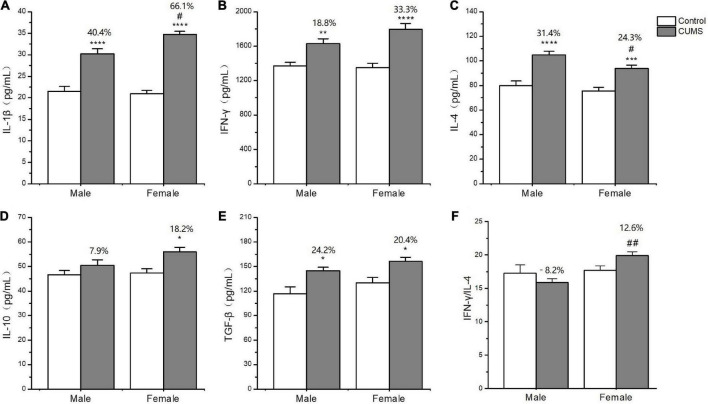
The changes of inflammation cytokines in serum of male and female rats. **(A–F)** The concentrations of inflammation cytokines of IL-1β, IFN-γ, IL-4, IL-10, and TGF-β, and the ratio of IFN-γ/IL-4 in serum. *p < 0.05, **p < 0.01, ***p < 0.001, and ****p < 0.0001 vs. control with same sex, ^#^*p* < 0.05, ^##^*p* < 0.01 vs. male stress group.

**FIGURE 8:**
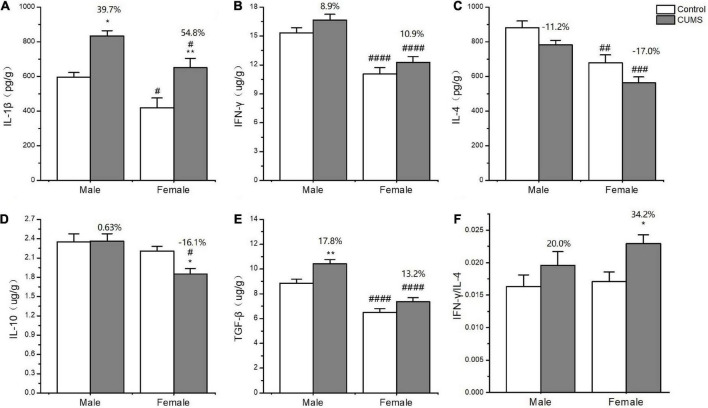
The changes of inflammation cytokines in the hippocampus of male and female rats. **(A–F)** The concentrations of inflammation cytokines of IL-1β, IFN-γ, IL-4, IL-10, and TGF-β, and the ratio of IFN-γ/IL-4 in the hippocampus. *p < 0.05, **p < 0.01 vs. control with same sex, ^#^*p* < 0.05, ^##^*p* < 0.01, ^###^*p* < 0.001, and ^####^*p* < 0.0001 vs. male in same control or CUMS group.

In the serum, two-way ANOVA indicated that CUMS significantly changed the concentration of IL-1β (CUMS: *F*_1, 56_ = 130.6, *p* < 0.0001), IFN-γ (CUMS: *F*_1, 56_ = 48.74, *p* < 0.0001), IL-4 (CUMS: *F*_1, 58_ = 47.68, *p* < 0.0001; *F*_1, 58_ = 4.934, *p* = 0.0302), and TGF-β (CUMS: *F*_1, 49_ = 15.11, *p* = 0.0003), while the concentration of IL-4 (*F*_1, 58_ = 5.877, *p* = 0.0185) and the ratio of IFN-γ/IL-4 (*F*_1, 56_ = 7.267, *p* = 0.0093) were influenced by sex. Moreover, the interaction between CUMS and sex significantly exerted the effects on IL-1β (*F*_1, 56_ = 4.040, *p* = 0.0493) and the ratio of IFN-γ/IL-4 (*F*(1, 56) = 4.844, *p* = 0.0319). The *post hoc* test showed that CUMS significantly increased the level of IL-1β (female: *p* < 0.0001; male: *p* < 0.0001), IFN-γ (female: *p* < 0.0001; male: *p* = 0.0034), IL-4 (female: *p* = 0.0005; male: *p* < 0.0001), and TGF-β (female: *p* = 0.0396; male: *p* = 0.0396) in the serum of both sexes. Greater increases in the IL-1β (*p* = 0.0430) and the IFN-γ/IL-4 ratio (*p* = 0.0055) and lower increases in the IL-4 (*p* = 0.0314) were observed in female rats than in male rats (Figures 7A–F). In the hippocampus, two-way ANOVA indicated that either CUMS or sex significantly affected IL-1β (CUMS: *F*_1, 31_ = 20.20, *p* < 0.0001; sex: *F*_1, 31_ = 12.33, *p* = 0.0014), IFN-γ (CUMS: *F*_1, 43_ = 4.852, *p* = 0.0330; sex: *F*_1, 43_ = 55.08, *p* < 0.0001), IL-4 (CUMS: *F*_1, 44_ = 9.655, *p* = 0.0033; *F*_1, 44_ = 37.12, *p* < 0.0001), and TGF-β (CUMS: *F*_1, 46_ = 17.80, *p* = 0.0001; sex: *F*_1, 46_ = 68.96, *p* < 0.0001). However, the *post hoc* test showed that hippocampal concentrations of IFN-γ (*p* < 0.0001), IL-4 (*p* = 0.0011), and TGF-β (*p* < 0.0001) were lower in female rats than in male rats under the control condition. CUMS markedly increased by 54.8% IL-1β concentration in female rats but 39.7% in male rats compared with sex-matched control (female: *p* = 0.0042; male: *p* = 0.0360). Moreover, the contents of the other proinflammatory cytokine IFN-γ (*p* < 0.0001) and the anti-inflammatory cytokines IL-10 (*p* = 0.0305), IL-4(*p* = 0.0002), and TGFβ (*p* < 0.0001) were also significantly lower in female rats than in male rats after exposure to CUMS (Figures 8A-F).

### Changes in the concentration of neurotransmitters and their metabolites in the hippocampus

**FIGURE 9:**
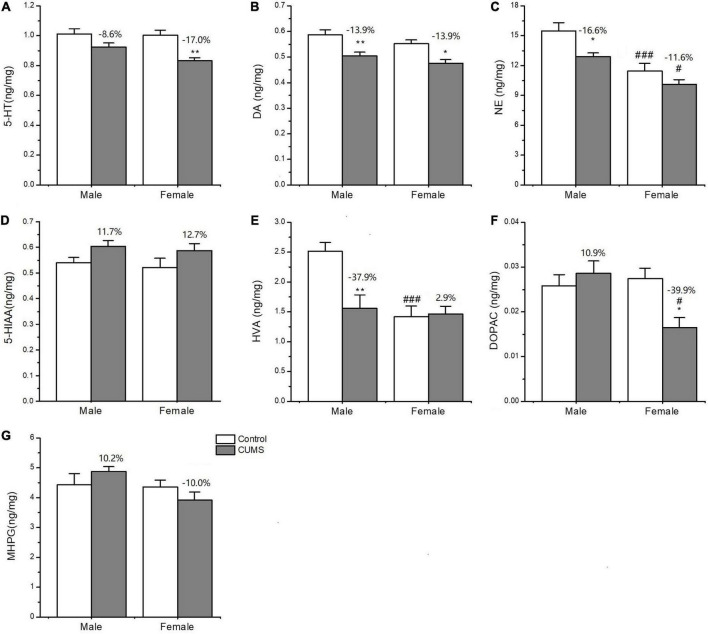
The concentrations of neurotransmitters and metabolites in the hippocampus of male and female rats. **(A–G)** The concentrations of neurotransmitters and metabolites detected in the hippocampus. 5-HT, DA, NE, 5-HIAA, HVA, DOPAC, and MPHG. *p < 0.05, **p < 0.01 vs. control with same sex, ^#^*p* < 0.05, ^###^*p* < 0.001 vs. male in same control or CUMS group.

Two-way ANOVA indicated that both CUMS and sex, or either of them significantly affected the concentration of neurotransmitters and metabolites, serotonin (5-HT) (CUMS: *F*_1, 24_ = 18.11, *p* = 0.0003), dopamine (DA) (CUMS: *F*_1, 24_ = 23.38, *p* < 0.0001), homovanillic acid (HVA) (*F*_1, 24_ = 6.894, *p* = 0.0148; sex: *F*_1, 24_ = 11.75, *p* = 0.0022), also known as 3,4-Dihydroxyphenylacetic acid (DOPAC) (sex: *F*_1, 24_ = 4.382, *p* = 0.0471), and norepinephrine (NE) (CUMS: *F*_1, 24_ = 9.282, *p* = 0.0056; sex: *F*_1, 24_ = 27.94, *p* < 0.0001). Moreover, the interaction between CUMS and sex significantly exerted the effects on DOPAC (*F*_1, 24_ = 7.644, *p* = 0.0108) and HVA (*F*_1, 24_ = 8.183, *p* = 0.0086). Moreover, CUMS significantly reduced the 5-HT content in female rats without changing in male rats (female: *p* = 0.0027; male: *p* = 0.1586) (Figures 9A, D). The dopamine (DA) content in the hippocampus was significantly reduced in rats of both sexes after exposure to stress (female: *p* = 0.0115; male: *p* = 0.0087). The concentration of DA metabolite DOPAC was significantly decreased in female rats than in male rats (*p* = 0.0129). For another metabolite of DA, homovanillic acid (HVA) concentration was lower in female rats than in male rats under the control condition (*p* = 0.0009), while the sex difference disappeared after stress (Figures 9B, E, F). Under the control condition, the concentration of the neurotransmitter norepinephrine (NE) was lower in females than males (*p* = 0.0009), but no sex difference was found in the 3-methoxy-4-hydroxyphenylglycol (MHPG) level. Moreover, CUMS reduced the NE content in rats of both sexes, and again, the content of which was lower in female rats than in male rats (*p* = 0.0219) (Figures 9C, G).

### Sex differences in the expression of glial markers, neurotrophic factors, and their receptors

**FIGURE 10:**
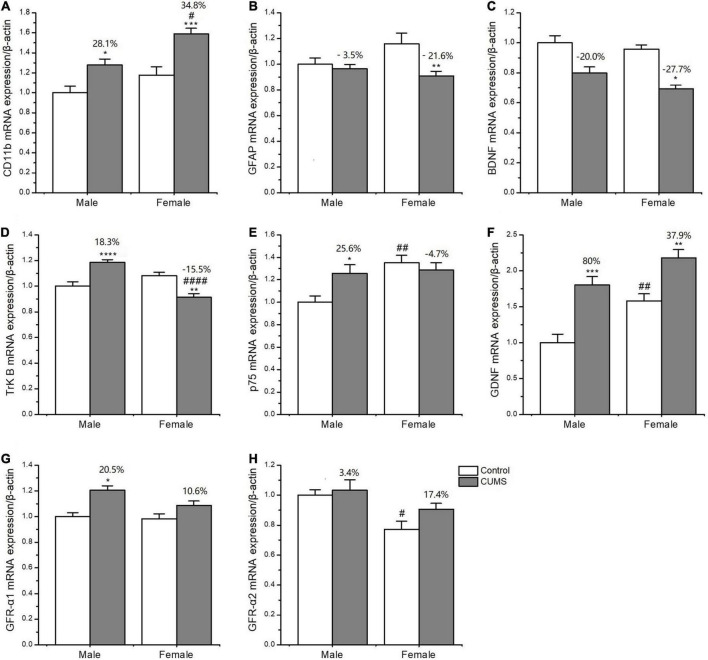
CUMS influenced the mRNA expression of microglial and astroglial marker genes in the hippocampus of male and female rats. **(A–H)** qPCR analysis of microglial and astroglial marker genes of CD11b, glial fibrillary acidic protein (GFAP), brain-derived neurotrophic factor (BDNF), tyrosine kinase B receptor (TrkB), p75, GDNF, GFR-α1, and GFR-α2. *p < 0.05, **p < 0.01, ***p < 0.001, and ****p < 0.0001 vs. control with same sex, ^#^*p* < 0.05, ^##^*p* < 0.01, ^####^*p* < 0.0001 vs. male in same control or CUMS group.

**FIGURE 11:**
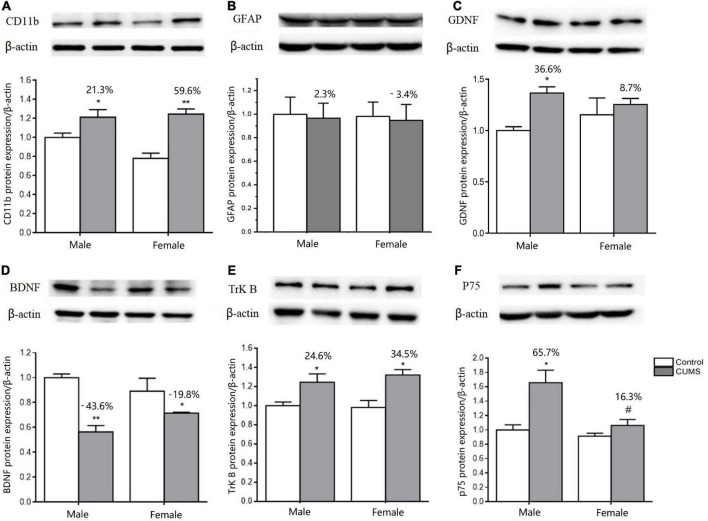
CUMS influenced the microglial and astroglial marker protein expressions in the hippocampus of male and female rats. **(A–F)** WB analysis of microglial and astroglial marker protein expression of CD11b, GFAP, GDNF, BDNF, TrK B, and p75. *p < 0.05, **p < 0.01 vs. control with same sex. ^#^*p* < 0.05 vs. male stress group.

Two-way ANOVA indicated that both CUMS and sex, or either of them significantly affected the mRNA transcriptions of CD11b (CUMS: *F*_1, 34_ = 22.48, *p* < 0.0001, sex: *F*_1, 34_ = 11.04, *p* = 0.0021), GFAP (CUMS: *F*_1, 36_ = 6.453, *p* = 0.0155), BDNF (CUMS: *F*_1, 32_ = 40.66, *p* < 0.0001; sex: *F*_1, 32_ = 4.189, *p* = 0.0490), TrK B (sex: *F*_1, 39_ = 9.638, *p* = 0.0035), P75 (sex: *F*_1, 35_ = 7.972, *p* = 0.0078), GDNF (CUMS: *F*_1, 20_ = 39.32, *p* < 0.0001; sex: *F*_1, 20_ = 18.62, *p* = 0.0003), GFR-α1 (CUMS: *F*_1, 41_ = 13.47, *p* = 0.0007), and GFR-α2 (sex: *F*_1, 32_ = 12.06, *p* = 0.0015). Furthermore, the interaction between CUMS and sex was significant in TrK B (*F*_1, 39_ = 38.23, *p* < 0.0001) and P75 (*F*_1, 35_ = 5.649, *p* = 0.0231). The *post hoc* test showed that there were no sex differences in the mRNA expression of CD11b under the control condition, which was largely upregulated in rats of both sexes (female: *p* = 0.0009, male: *p* = 0.0403) after exposure to stress. The increasing percent was about 34.8% in female rats and 28.1% in male rats, and a similar change trend was detected in CD11b protein with a 59.6% increase in the female rats and 21.3% in the male rats (Figures 10A, 11A). CUMS downregulated the mRNA level of GFAP in the female rats without affecting it in the male rats (female: 0.0095; male: *p* = 0.7182). However, no change was observed in GFAP protein expression (Figures 10B, 11B). CUMS significantly reduced mRNA expression of BDNF in female rats (*p* = 0.0402) with 27.7%, but decreased BDNF protein was 19.8% in female rats to 43.6% in male rats (Figures 10C, 11D). With regard to the expression of two BDNF receptors, no sex difference was observed in the mRNA and protein expression of TrK B under control conditions, while the mRNA level was markedly increased in the male rats and decreased in the female rats after exposure to CUMS (female: *p* = 0.0025; male: *p* < 0.0001; Figure 10D). Moreover, CUMS increased the percentage of TrK B protein to 24.6% in male rats and 34.5% in female rats (Figure 11E). The mRNA expression of another receptor, P75, was higher in female rats than in males under control conditions (*p* = 0.0032) without sex differences after exposure to CUMS (Figure 10E). Although no sex difference was found in P75 protein expression under the control condition, it was much lower in female rats than in males under CUMS condition (*p* = 0.0186) (Figure 11F).

For another neurotrophin GDNF and its receptors, the mRNA expression of GDNF was much higher (*p* = 0.0472), and GFR-α2 was lower (*p* = 0.0133) in control females than control males, without sex difference in GFR-α1 expression. Compared with the baseline level in the control condition, the mRNA levels of GDNF were more significantly elevated in male rats by 80% than in female rats by 37.9% after exposure to CUMS (Figures 10F–H). Consistently, the protein expression of GDNF was also more significantly increased in male rats at 36.6% and in female rats at 8.7% after exposure to CUMS (Figure 11C).

## Discussion

The present study compared sex differences in behaviors as well as sex differences in the underlying mechanisms involved in corticosteroid secretion, glial overactivation–mediated inflammatory responses, and monoamine neurotransmitter defects in a CUMS-induced model of depression. The findings gained from the study are discussed below.

First, CUMS induced depression- and anxiety-like behaviors in both male and female rats with sex-specific sensitivities, which were partly due to sex differences under control conditions. Female rats gained less body weight than male rats under control conditions, which indicated that male rats were originally stronger than female rats. Under CUMS conditions, the decreased sucrose preference and increased immobility times in the FST were more pronounced in female rats than in male rats when compared to their respective baselines; these results suggested that the female rats were more vulnerable to stress. However, the results from the OFT and EPM showed that CUMS induced greater anxiety-like behavior in male rats than in female rats. For example, male rats showed a lower ratio of time spent in and the number of entries into the open arms/closed arms in the EPM test and less time spent in the central zone in the OFT than female rats. The finding of the present study is that proinflammatory IL-1β concentration was higher in male rat hippocampus than in females, which might contribute to more anxious behavior in male rats since the rodents received peripheral or central IL-1β administration displayed anxiogenic behavior (Rossi et al., 2012). With regard to more depression-like behaviors in females, the hyper-active HPA axis may take responsibility (Keller et al., 2017; Thomson and Craighead, 2018). The present study showed that CUMS elevated serum corticosterone concentrations to a greater extent in female rats than in male rats. The same change was previously reported in both animal (Eid et al., 2019) and human studies (Kokras et al., 2019), indicating that the HPA axis in females is more sensitive to stress stimuli (Ma et al., 2019).

According to the macrophage/T-lymphocyte hypothesis of depression, the overproduction of proinflammatory cytokines by macrophages and lymphocyte subtypes, such as Th1 cell–derived factors or chronic stress–induced glucocorticoids, can cause neuroinflammation and depression. The present study compared the changes in peripheral cytokine levels between male and female rats, which showed that CUMS largely increased serum IL-1β concentrations and decreased serum IL-4 concentrations, leading to a higher IFN-γ/IL-4 ratio in female rats than in male rats. Similar to the trend observed in the periphery, the increase in the IFN-γ/IL-4 ratio in the hippocampus was found in female rats. These data indicated that an imbalance between Th1 and Th2 responses occurred in female rats. Consistently, a previous study reported a trend of enhanced Th1 responses, as shown by an IFN-γ/IL-4 ratio, which was more obvious in female patients with depression (Myint et al., 2005). The most powerful anti-inflammatory cytokine IL-10 from Th2 was reported to perform an antidepressant effect by promoting neuronal survival and inhibiting the death of glial cells (Roque et al., 2009). Moreover, TGFβ also displayed neuroprotective effects and synaptic plasticity against depression (Qiu et al., 2021). The present study, for the first time, found the concentration of TGFβ was originally lower in the female hippocampus. More interestingly, CUMS significantly reduced the level of IL-10 in female rats but significantly elevated the level of TGFβ in male rats. These findings further explain why females are more susceptible to depression.

Regarding microglia phenotype in rats of different sexes, the present study found that CUMS polarized microglia toward the M1 phenotype by increasing CD11b mRNA and protein expression. Since both Th1 activation (Prajeeth et al., 2014) and glucocorticoid overproduction can trigger microglial M1 polarization (Picard et al., 2021), the CUMS-induced increase in CD11b expression might result from the higher Th1 response and higher corticosterone concentrations in female rats. As a consequence of M1 microglia activation, astrocyte function may be suppressed. Indeed, CUMS significantly decreased the mRNA and protein expression of BDNF and caused a similar change in TrK B protein expression in rats of both sexes. BDNF is known to promote neurogenesis, neuronal survival, and synaptic plasticity when combined with its receptor TrKB (Numakawa and Odaka, 2021). Thus, CUMS-induced alterations in BDNF function in female and male rats might be a common mechanism that contributes to depressive behaviors. More importantly, the present study found some sex-specific changes: (1) CUMS significantly decreased the mRNA expression of TrkB in female rats compared to male rats; (2) CUMS significantly upregulated p75 expression in male rats but not in the females; (3) CUMS upregulated the mRNA and protein expression of GDNF more significantly in male rats than in female rats, which was accompanied a trend of higher GFR-α1 and GFR-α2 mRNA expression. Being opposite to TrKB receptor function, P75 was reported to induce cell apoptosis by binding to pro-BDNF when TrKB or BDNF expression was reduced (Kenchappa et al., 2010). However, amazingly, P75 can increase the expression of GDNF genes to protect against and repair stress-induced neuronal injury (Steljes et al., 1999; Barati et al., 2006). Therefore, the upregulations of P75 and GDNF at the same time in the male rats may play a neuroprotective role against stress-induced depression, which deserves further study. Furthermore, the lower expression of GDNF and its receptors in females may be another factor that contributes to female vulnerability to the onset of depression.

As another consequence of M1 microglia activation, the excessive release of proinflammatory cytokines may damage neurotransmitter function and neuroplasticity through astrocyte dysfunction, which results in monoamine synaptic deficits (Yohn et al., 2016) and glutamate neurotoxicity (Haroon et al., 2017). The reduction in NE and 5-HT is the hallmark of major depression (Abdel-Bakky et al., 2021). NE can inhibit the transcription of proinflammatory cytokines and promote the production of neurotrophic factors (O’Donnell et al., 2012). The important findings in the present study were lower concentrations of NE and its metabolite MHPG in the hippocampus of female rats than those in male rats under both control and stress conditions. CUMS also reduced the concentrations of another important monoamine neurotransmitter, namely, 5-HT, which effects were more pronounced in female rats than in male rats in this study. The deficiencies in NE and 5-HT concentrations under original and CUMS conditions may contribute to the higher prevalence of depression in females. Moreover, dysfunction of the DA system in the brain also induces the onset of depressive behaviors, such as anhedonia and lack of motivation (Felger and Treadway, 2017). The present study showed that CUMS notably decreased the DA levels in the hippocampus of the rats of both sexes, and no sex difference was observed. However, a lower HVA concentration was observed in female rats than in male rats under control conditions. CUMS then significantly decreased the DOPAC concentration in female rats without affecting the male rats. Because a lower level of HVA has been reported in patients with depression (Ogawa and Kunugi, 2019), lower levels of HVA may be implicated in the higher risk of depression in females, which needs further study.

## Conclusion

In summary, this study first showed that under control conditions, female rats showed a lower body weight gain and a higher Th1/Th2 cytokine ratio in the hippocampus, which were associated with NE and HVA deficiency. Additionally, the higher expression of the neurotrophic factor GDNF and the BDNF receptor P75 was observed in female rats than in male rats. Second, the present study found that CUMS induced more significant depression-like behaviors, such as anhedonia, immobility, and exploration behaviors in female rats than in male rats. The mechanisms underlying the more depression-like behaviors in female rats after exposure to stress mainly include increased GC levels, imbalanced Th1/Th2 cytokine levels in the periphery and hippocampus, greater enhanced polarization of microglia to the M1 phenotype, and more pronounced deficiency of 5-HT and NE. These findings provide evidence to explain why females suffer worse depression than males, at least in stressful environments.

## Data availability statement

The original contributions presented in this study are included in the article/supplementary material, further inquiries can be directed to the corresponding author.

## Ethics statement

This animal study was reviewed and approved by Local Bioethics Committee of Guangdong Ocean University.

## Author contributions

CS applied for the grant, directed and designed the experiments, and edited the manuscript. JX and HW performed most of the experiments, analyzed the data, and wrote the manuscript. CZ, BL, YL, KL, and PL performed a part of the experiments. All authors read and agreed to the published version of the manuscript.

## References

[B1] Abdel-Bakky M. Amin E. Abdellatif A. (2021). Mental depression: Relation to different disease status, newer treatments and its association with COVID-19 pandemic (Review). *Mol. Med. Rep.* 24:839. 10.3892/mmr.2021.12479 34633054 PMC8524409

[B2] Banasr M. Valentine G. Li X. Gourley S. Taylor J. Duman R. (2007). Chronic unpredictable stress decreases cell proliferation in the cerebral cortex of the adult rat. *Biol. Psychiatry* 62 496–504. 10.1016/j.biopsych.2007.02.006 17585885

[B3] Barati S. Hurtado P. Zhang S. Tinsley R. Ferguson I. Rush R. A. (2006). GDNF gene delivery via the p75(NTR) receptor rescues injured motor neurons. *Exp. Neurol.* 202 179–188. 10.1016/j.expneurol.2006.05.027 16842780

[B4] Cavanagh A. Wilson C. Kavanagh D. Caputi P. (2017). Differences in the expression of symptoms in men versus women with depression: A systematic review and meta-analysis. *Harv. Rev. Psychiatry* 25 29–38. 10.1097/HRP.0000000000000128 28059934

[B5] Christmas D. M. Potokar J. Davies S. J. (2011). A biological pathway linking inflammation and depression: Activation of indoleamine 2, 3-dioxygenase. *Neuropsychiatr. Dis. Treat.* 7 431–439. 10.2147/NDT.S17573 21792309 PMC3140295

[B6] Eid R. Gobinath A. Galea L. (2019). Sex differences in depression: Insights from clinical and preclinical studies. *Prog. Neurobiol.* 176 86–102. 10.1016/j.pneurobio.2019.01.006 30721749

[B7] Felger J. Treadway M. (2017). Inflammation effects on motivation and motor activity: Role of dopamine. *Neuropsychopharmacology* 42 216–241. 2016.143 10.1038/npp.2016.143 27480574 PMC5143486

[B8] Haroon E. Miller A. Sanacora G. (2017). Inflammation, glutamate, and glia: A trio of trouble in mood disorders. *Neuropsychopharmacology* 42 193–215. npp.2016.199 10.1038/npp.2016.199 27629368 PMC5143501

[B9] Hu C. Luo Y. Wang H. Kuang S. Liang G. Yang Y. (2017). Re-evaluation of the interrelationships among the behavioral tests in rats exposed to chronic unpredictable mild stress. *PLoS One* 12:e0185129. 10.1371/journal.pone.0185129 28931086 PMC5607203

[B10] Keller J. Gomez R. Williams G. Lembke A. Lazzeroni L. Murphy G. (2017). axis in major depression: Cortisol, clinical symptomatology and genetic variation predict cognition. *Mol. Psychiatry* 22 527–536. 10.1038/mp.2016.120 27528460 PMC5313380

[B11] Kenchappa R. Tep C. Korade Z. Urra S. Bronfman F. Yoon S. (2010). p75 neurotrophin receptor-mediated apoptosis in sympathetic neurons involves a biphasic activation of JNK and up-regulation of tumor necrosis factor-alpha-converting enzyme/ADAM17. *J. Biol. Chem.* 285 20358–20368. 10.1074/jbc.M109.082834 20421303 PMC2888447

[B12] Kessler R. (2013). Epidemiology of women and depression. *J. Affect. Disord.* 74 5–13. 10.1016/s0165-0327(02)00426-3 12646294

[B13] Kokras N. Hodes G. Bangasser D. Dalla C. (2019). Sex differences in the hypothalamic-pituitary-adrenal axis: An obstacle to antidepressant drug development? *Br. J. Pharmacol.* 176 4090–4106. 10.1111/bph.14710 31093959 PMC6877794

[B14] Kraeuter A. Guest P. Sarnyai Z. (2019). The elevated plus maze test for measuring anxiety-like behavior in rodents. *Methods Mol. Biol.* 1916 69–74. 10.1007/978-1-4939-8994-24 30535682

[B15] Li P. Zhang F. Li Y. Zhang C. Yang Z. Zhang Y. (2021). Isoginkgetin treatment attenuated lipopolysaccharide-induced monoamine neurotransmitter deficiency and depression-like behaviors through downregulating p38/NF-κB signaling pathway and suppressing microglia-induced apoptosis. *J. Psychopharmacol.* 35 1285–1299. 10.1177/02698811211032473 34281416 PMC8521360

[B16] Ma L. Xu Y. Wang G. Li R. (2019). What do we know about sex differences in depression: A review of animal models and potential mechanisms. *Prog. Neuropsychopharmacol. Biol. Psychiatry* 89 48–56. 10.1016/j.pnpbp.2018.08.026 30165122

[B17] Maes M. Smith R. Scharpe S. (1995). The monocyte-T-lymphocyte hypothesis of major depression. *Psychoneuroendocrinology* 20 111–116. 10.1016/0306-4530(94)00066-j 7899532

[B18] Moraes C. Zaverucha-do-Valle C. Fleurance R. Sharshar T. Bozza F. d’Avila J. (2021). Neuroinflammation in sepsis: Molecular pathways of microglia activation. *Pharmaceuticals (Basel)* 14:416. 10.3390/ph14050416 34062710 PMC8147235

[B19] Myint A. Leonard B. Steinbusch H. Kim Y. (2005). Th1, Th2, and Th3 cytokine alterations in major depression. *J. Affect. Disord.* 88 167–173. 2005.07.008 10.1016/j.jad 16126278

[B20] Numakawa T. Odaka H. (2021). Brain-derived neurotrophic factor signaling in the pathophysiology of Alzheimer’s disease: Beneficial effects of flavonoids for neuroprotection. *Int. J. Mol. Sci.* 22:5719. 10.3390/ijms22115719 34071978 PMC8199014

[B21] O’Donnell J. Zeppenfeld D. McConnell E. Pena S. Nedergaard M. (2012). Norepinephrine: A neuromodulator that boosts the function of multiple cell types to optimize CNS performance. *Neurochem. Res.* 37 2496–2512. 10.1007/s11064-012-0818-x 22717696 PMC3548657

[B22] Ogawa S. Kunugi H. (2019). Evidence for reduced homovanillic acid (HVA) in the cerebrospinal fluid of patients with depression. *J. Affect. Disord.* 255 S0165–S0327. 10.1016/j.jad.2019.04.028 31006502

[B23] Peng Z. Zhang C. Yan L. Zhang Y. Yang Z. Wang J. (2020). EPA is more effective than DHA to improve depression-like behavior, glia cell dysfunction and hippcampal apoptosis signaling in a chronic stress-induced rat model of depression. *Int. J. Mol. Sci.* 21:1769. 10.3390/ijms21051769 32150824 PMC7084382

[B24] Picard K. Bisht K. Poggini S. Garofalo S. Golia M. Basilico B. (2021). Microglial-glucocorticoid receptor depletion alters the response of hippocampal microglia and neurons in a chronic unpredictable mild stress paradigm in female mice. *Brain Behav. Immun.* 97 423–439. 10.1016/j.bbi.2021.07.022 34343616

[B25] Prajeeth C. Löhr K. Floess S. Zimmermann J. Ulrich R. Gudi V. (2014). Effector molecules released by Th1 but not Th17 cells drive an M1 response in microglia. *Brain Behav. Immun.* 37 248–259. 10.1016/j.bbi.2014.01.001 24412213

[B26] Qiu A. Zhang H. Wang C. Chong Y. Shek L. Gluckman P. (2021). Canonical TGF-β signaling regulates the relationship between prenatal maternal depression and amygdala development in early life. *Transl. Psychiatry* 11:170. 10.1038/s41398-021-01292-z 33723212 PMC7961018

[B27] Roque S. Correia-Neves M. Mesquita A. Palha J. Sousa N. (2009). Interleukin-10: A key cytokine in depression? *Cardiovasc. Psychiatry Neurol.* 2009:187894. 10.1155/2009/187894 19936104 PMC2775686

[B28] Rossi S. Sacchetti L. Napolitano F. De Chiara V. Motta C. Studer V. (2012). Interleukin-1β causes anxiety by interacting with the endocannabinoid system. *J. Neurosci.* 32 13896–13905. 10.1523/JNEUROSCI.1515-12.2012 23035099 PMC6704788

[B29] Seney M. Huo Z. Cahill K. French L. Puralewski R. Zhang J. (2018). Opposite molecular signatures of depression in men and women. *Biol. Psychiatry* 84 18–27. 10.1016/j.biopsych.2018.01.017 29548746 PMC6014892

[B30] Smith R. (1991). The macrophage theory of depression. *Med. Hypotheses* 35 298–306. 10.1016/0306-9877(91)90272-z 1943879

[B31] Steljes T. Kinoshita Y. Wheeler E. Oppenheim R. von Bartheld C. (1999). Neurotrophic factor regulation of developing avian oculomotor neurons: Differential effects of BDNF and GDNF. *J. Neurobiol.* 41 295–315. 10512985

[B32] Thomson F. Craighead M. (2018). Innovative approaches for the treatment of depression: Targeting the HPA axis. *Neurochem. Res.* 33 691–707. 10.1007/s11064-007-9518-3 17960478

[B33] Turnbull A. Rivier C. (1995). Regulation of the HPA axis by cytokines. *Brain Behav. Immun.* 9 253–275. 10.1006/brbi.1995.1026 8903845

[B34] Willner P. Towell A. Sampson D. Muscat R. (1987). Reduction of sucrose preference by chronic unpredictable mild stress, and its restoration by a tricyclic antidepressant. *Psychopharmacology (Berl)* 93 358–364. 10.1007/BF00187257 3124165

[B35] Yan L. Gu M. Yang Z. Xia J. Li P. Vasar E. (2021). Endogenous n-3 PUFAs attenuated olfactory bulbectomy-induced behavioral and metabolomic abnormalities in Fat-1 mice. *Brain Behav. Immun.* 96 143–153. 10.1016/j.bbi.2021.05.024 34052364

[B36] Yang C. Hawkins K. Doré S. Candelario-Jalil E. (2019). Neuroinflammatory mechanisms of blood-brain barrier damage in ischemic stroke. *Am. J. Physiol. Cell Physiol.* 316 C135–C153. 10.1152/ajpcell.00136.2018 30379577 PMC6397344

[B37] Yohn S. Arif Y. Haley A. Tripodi G. Baqi Y. Müller C. (2016). Effort-related motivational effects of the pro-inflammatory cytokine interleukin-6: Pharmacological and neurochemical characterization. *Psychopharmacology (Berl)* 233 3575–3586. 10.1007/s00213-016-4392-9 27497935

[B38] Zhang C. Kalueff A. Song C. (2019a). Minocycline ameliorates anxiety-related self-grooming behaviors and alters hippocampal neuroinflammation, GABA and serum cholesterol levels in female Sprague-Dawley rats subjected to chronic unpredictable mild stress. *Behav. Brain Res.* 363 109–117. 10.1016/j.bbr.2019.01.045 30703398

[B39] Zhang C. Zhang Y. Li Y. Liu B. Wang H. Li K. (2019b). Minocycline ameliorates depressive behaviors and neuro-immune dysfunction induced by chronic unpredictable mild stress in the rat. *Behav. Brain Res.* 356 348–357. 2018.07.001 10.1016/j.bbr 30003978

